# Colour-Temperature Correspondences: When Reactions to Thermal Stimuli Are Influenced by Colour

**DOI:** 10.1371/journal.pone.0091854

**Published:** 2014-03-11

**Authors:** Hsin-Ni Ho, George H. Van Doorn, Takahiro Kawabe, Junji Watanabe, Charles Spence

**Affiliations:** 1 NTT Communication Science Laboratories, Nippon Telegraph and Telephone Corporation, Atsugi, Kanagawa, Japan; 2 School of Applied Media and Social Sciences, Federation University, Churchill, Victoria, Australia; 3 Crossmodal Research Laboratory, Department of Experimental Psychology, University of Oxford, Oxford, United Kingdom; Université catholique de Louvain, Belgium

## Abstract

In our daily lives, information concerning temperature is often provided by means of colour cues, with red typically being associated with warm/hot, and blue with cold. While such correspondences have been known about for many years, they have primarily been studied using subjective report measures. Here we examined this correspondence using two more objective response measures. First, we used the Implicit Association Test (IAT), a test designed to assess the strength of automatic associations between different concepts in a given individual. Second, we used a priming task that involved speeded target discrimination in order to assess whether priming colour or thermal information could invoke the crossmodal association. The results of the IAT confirmed that the association exists at the level of response selection, thus indicating that a participant’s responses to colour or thermal stimuli are influenced by the colour-temperature correspondence. The results of the priming experiment revealed that priming a colour affected thermal discrimination reaction times (RTs), but thermal cues did not influence colour discrimination responses. These results may therefore provide important clues as to the level of processing at which such colour-temperature correspondences are represented.

## Introduction

Indicating the likely temperature of an object by means of colour (red = warm/hot; blue = cool/cold) is common practice in the fields of industrial and interior design in many parts of the world [1]–[7]. Just think, for example, about the colour scheme used on hot and cold taps. While crossmodal correspondences between colour and temperature have long been reported, they have typically been studied by means of subjective measures. Previously, participants have been instructed to rate colour patches or coloured stimuli as being either warm or cool [5], [8]–[13] or, conversely, they have been presented with thermal stimuli and asked to report which colour they were reminded of [10]. While earlier studies have typically relied on subjective reports in order to establish the existence of a crossmodal correspondence between colour and temperature, here we utilized a pair of more objective behavioural measures: Namely, a simplified version of the Implicit Association Test (IAT) and a crossmodal priming task involving speeded target discrimination.

The IAT is designed to assess the strength of the association at the level of response selection [14]. In this task, participants are instructed to respond as rapidly and accurately as possible to a series of unimodal target stimuli, using two response keys. By now, a large body of empirical research has demonstrated that participants’ response latencies tend to be faster when the two stimuli assigned to a given response key are associated with one another (congruent), as compared to the RTs seen in those conditions in which unrelated, or incongruent, stimuli are assigned to the same response key [14]. A simplified version of this paradigm has been introduced recently by Parise and Spence [15]. In the experiment reported here, two stimuli (one colour patch and one thermal word) were assigned to each response key in each block of experimental trials. The assignment of the colour and thermal stimuli to each response key was manipulated so that half of the experimental blocks were assumed to be congruent (e.g., red and warm), while the other half were considered incongruent (e.g., red and cold). Differences in RTs between different stimulus-response key assignments are taken as evidence of the existence of a compatibility effect.

The priming study was designed to examine the impact of priming on the processing of a target stimulus. In the experiment reported here, the prime and target stimuli were presented sequentially with an interstimulus interval of 2,000 ms, and the participants had to perform a speeded discrimination of the target stimulus. The colours were presented as colour patches, while the thermal stimuli were delivered via thermal words or by means of actual thermal stimuli (either warm or cold). We manipulated the combination of colour and thermal stimuli presented in each trial, so that half of the trials were congruent (e.g., red and warm) while the remainder were incongruent (e.g., red and cold). The prediction here was that priming by the presentation of a stimulus in one modality (e.g., red) might facilitate response latencies to identify a congruent target stimulus (e.g., a warm target) relative to an incongruent stimulus (e.g., a cold target).

The IAT and the crossmodal priming task are amongst the most commonly used paradigms for those researchers wanting to study crossmodal congruency effects (see [16] for a review). Although both tasks use RT as the dependent measure, they are different in several ways. For example, the number of stimuli presented in each trial differ. While only a single stimulus is presented on each trial in the IAT, two stimuli are presented in each trial in the priming task. The IAT therefore allows participants to focus their attention on the target stimulus and excludes any possible role of crossmodal interactions between the colour and thermal stimuli during perceptual processing. This characteristic of the IAT ensures that the effects reported in studies using this paradigm result from the difference in the compatibility between the various stimulus-response key assignments utilized. On the other hand, by presenting a prime and a target in a single trial, the priming task allows for the possible perceptual interaction between the colour and thermal stimuli during information processing. This task therefore assesses the influence of a priming stimulus on the perception and discrimination of the target stimulus. Thus, the level of crossmodal correspondence that the two paradigms examine is somewhat different; the IAT measures the strength of any association between two stimuli at the level of response selection; whereas the priming experiment, in essence, measures an ability to selectively attend to one source of sensory information while ignoring the influence of the other stimulus presented somewhat earlier in time. In the present study, our interest was in trying to determine whether the impact of colour-temperature correspondences would be different at these two distinct levels of information processing.

## Experiment 1

The purpose of this experiment was to investigate whether the crossmodal correspondence between colour and temperature could be demonstrated using the IAT.

### Materials and Methods

#### Participants

Eleven participants (six females) took part in this experiment. The participants had a mean age of 29.9 years (*SD* = 5.0 years, range 19–37 years). All of the participants had normal or corrected-to-normal vision and none of them reported any visual, motor, or neurological abnormalities. The experiment was approved by the ethics committee of NTT Communication Science Laboratories and conducted in accordance with the ethical standards in the 1964 Declaration of Helsinki. The participants gave their written informed consent before the start of the experiment.

#### Apparatus and materials

Experiment 1 was conducted in the NTT Communication Science Laboratories, Japan. The presentation of the stimuli and the collection of the responses were controlled by means of a computer (Mac Pro) running Matlab 7.5 with PsychToolbox 3 [17], [18]. The participants were seated in front of a 21″ CRT computer monitor with a resolution of 1024×768 pixels and a refresh rate of 60 Hz. The distance between the participant’s eyes and the screen was approximately 60 cm. The experiment was conducted in a dark and quiet room.

#### Stimuli

Two colour patches and two thermal words were used as stimuli in this experiment. The colour patches were 2.2×2.2 deg red and blue squares in RGB colour space (red square: 114, 0, 0; blue square: 0, 0, 255). The thermal words were 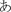 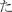 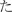 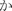 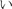 (which can be translated as “warm”) and 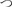 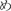 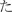 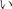 (which translates as “cold”) presented in 20 pt Sans-serif font. The colour of the texts was gray in RGB colour space (15, 20, 21). The luminance of the colour patches and the text were matched at 13 cd/m^2^. Each of the colour patches and words was presented at the centre of a black background subtending 35.8×26.9 deg.

#### Procedure

**Figure 1:**
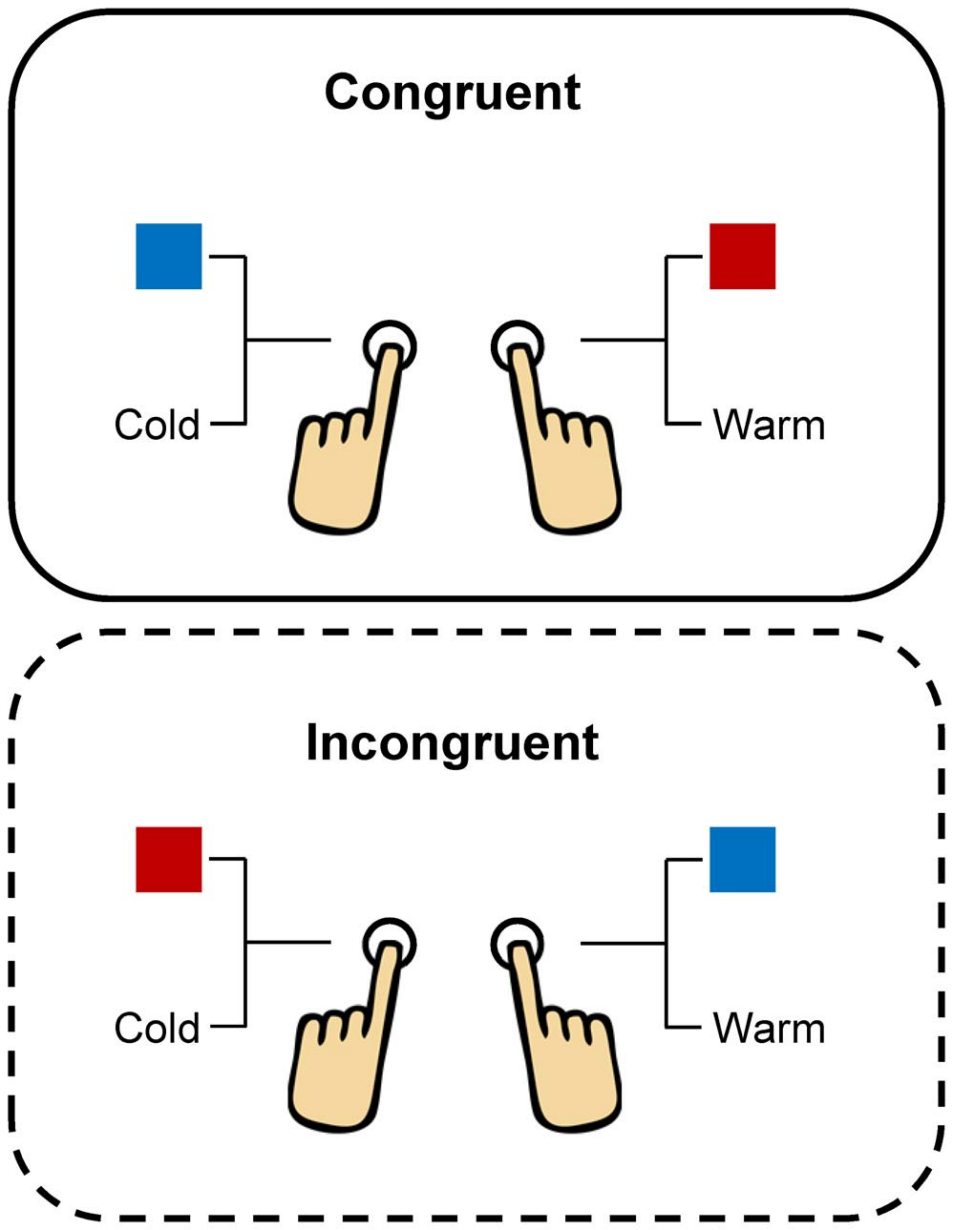
The stimulus-response key assignment. Congruent and incongruent assignments are illustrated in the upper and lower panels, respectively. Note that the locations (right or left) of the response keys were counterbalanced during the course of the experiment.

The procedure utilized here was identical to that reported by Parise and Spence [15]. Specifically, the participants were instructed to maintain their fixation on the centre of the screen and to respond to the target stimuli as rapidly and accurately as possible, by pressing one of two response keys (the ‘z’ and ‘m’ keys) on a computer keyboard. The stimulus-response key assignment is shown in Figure 1.

Each trial began with the presentation of a fixation point in the centre of the screen for a randomized interval of 500–600 ms. The removal of the fixation point was followed by a random interstimulus interval of 300–400 ms. Next, the target stimulus, either a colour patch (red or blue) or a thermal word (warm or cold), was presented. The stimulus remained on the screen for 300 ms before being removed. An auditory beep was provided as feedback whenever the participant made an incorrect response.

Each of the four response key assignments (congruent or incongruent, counterbalanced for left and right locations) was repeated six times giving rise to a total of 24 randomly alternating blocks of trials. Each block consisted of 20 trials (with each stimulus being repeated five times). Participants thus completed a total of 480 experimental trials. Each block was preceded by a one minute training phase to learn the key assignment. The 24 blocks were divided into three experimental sessions and each session lasted for approximately 20 min. The participants were allowed to take a 15 min rest between the sessions. The RT and accuracy of participants’ responses were collected.

### Results

RTs from the correct trials were used in the analysis (note that an average of 2.5% of the trials were removed from the RT analysis due to the participants’ responding incorrectly). The median of the RTs was calculated and the medians from the 11 participants were averaged to obtain the group mean.

**Figure 2:**
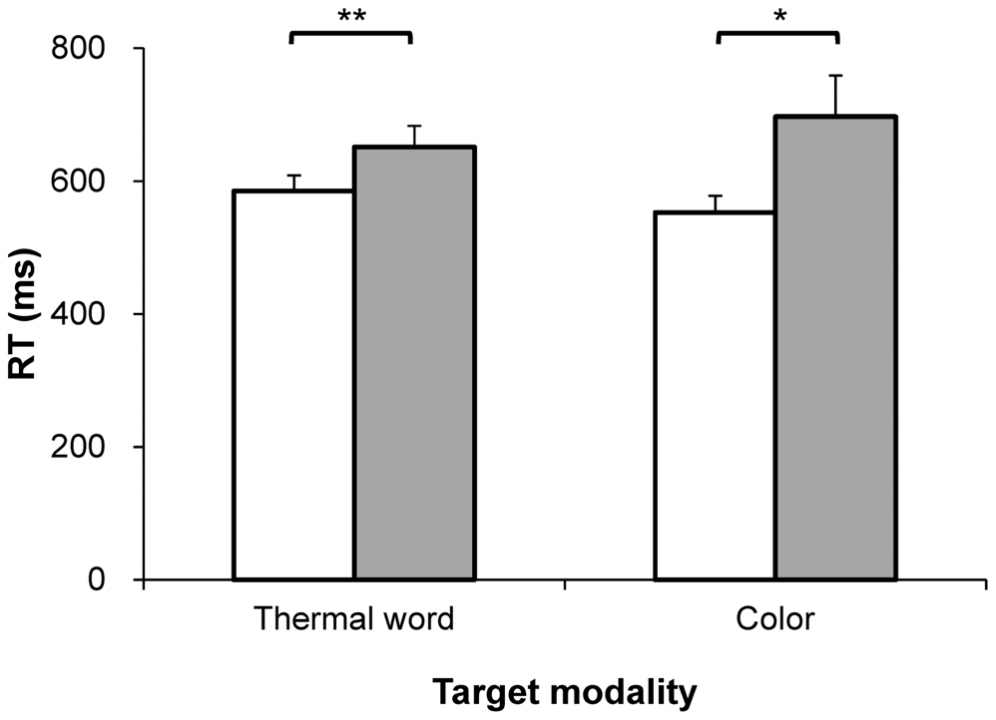
Results of the IAT task. RTs when participants discriminated the thermal words or colours in the IAT task (Experiment 1). Congruent key assignments are shown in white, incongruent assignments in gray. The error bars show the standard errors of the means. * and ** indicate statistical significance of *p*<.05 and *p*<.01, respectively (paired two-tailed *t* test).

Group mean RTs for the thermal word and colour targets are shown in Figure 2. Using the RT as a dependent variable, we conducted a two-way repeated-measures analysis of variance (ANOVA) with the target modality (thermal word/colour) and congruency of the key assignments (congruent/incongruent) as the within-participants factors. There was no main effect of the target modality [*F*(1,10)<1, *n*.*s*.]; in other words, RTs did not differ significantly for the two target modalities. There was a significant main effect of the congruency of the key assignment [*F*(1,10) = 12.69, *p* = .005]. Participants’ RTs were significantly shorter with the congruent key assignments (red-warm, blue-cold) than with the incongruent key assignments (red-cold, blue-warm). There was a significant interaction between the target modality and the congruency of the key assignment [*F*(1,10) = 5.88, *p* = .036]. To understand the nature of this interaction, we tested for simple main effects between two target modalities under congruent and incongruent conditions. The results indicate that for congruent conditions, colour targets led to shorter RTs than thermal word targets [*F*(1,10) = 15.45, *p*<.01]. For incongruent conditions, target modality has no significant effect on performance [*F*(1,10) = 1.52, *p* = 0.25].

## Experiment 2

The purpose of Experiment 2 was to determine whether the correspondence between colour and temperature would impact on response latencies in a simple priming task. In this experiment, colour and thermal stimuli were presented sequentially on each trial with the first stimulus acting as the task-irrelevant prime and the second stimulus as the target. When the prime consisted of a colour patch, the target was a thermal stimulus, and vice versa. The attributes of the prime were either putatively congruent with the target (e.g., red and warm) or else incongruent with it (e.g., red and cold). The colours were presented with colour patches and the thermal stimuli were delivered by means of thermal words (Experiment 2A) and physical thermal stimuli (Experiment 2B). The expectation was that participants would respond significantly more rapidly when stimulus combinations were congruent as compared to when they were incongruent [19]. It is of interest to know whether this effect would be different for semantically-presented versus physically-presented thermal stimuli.

### Materials and Methods

#### **Experiment 2A: Thermal word stimuli**

##### **Participants**

Fourteen participants (11 females) took part in the study. The participants had a mean age of 28.8 years (*SD* = 6.7 years, range 21–40 years). All of the participants had normal or corrected-to-normal vision and none reported any motor or neurological abnormalities. The experiment was approved by the ethics committee of NTT Communication Science Laboratories and conducted in accordance with the ethical standards in the 1964 Declaration of Helsinki. The participants gave their written informed consent before the start of the experiment.

##### **Apparatus and materials**

The apparatus and materials used in this experiment were the same as those used in Experiment 1.

##### **Stimuli**

The stimuli used in this experiment are the same as those used in Experiment 1. For each trial, the location of the prime was randomly determined to be either slightly above or slightly below the centre of a black background subtending 35.8×26.9 deg, while the target was situated in the opposing location (e.g., above centre if the prime was below centre).

##### **Procedure**

Each of the four prime/target pairings (red/warm, blue/cold, red/cold, and blue/warm) was repeated forty times giving rise to a total of 160 trials for each response target (colour patch or thermal word). The order of presentation of the trials was randomized for each participant, although the type of response target to be discriminated (colour patch or thermal word) was tested in a blocked manner in order to avoid any confusion about which stimulus the participants were to respond to. The order in which the blocks were presented was counterbalanced across participants. Each block lasted for approximately 15 minutes and participants were allowed to take a 15 minute rest at the end of each block.

1. *Thermal word discrimination task.* Each trial began with the presentation of a fixation point in the centre of the screen for a randomized interval of 500–600 ms. The removal of the fixation point was followed by a random interstimulus interval of 300–400 ms. Next, the priming colour patch (e.g., red) was presented for 2,000 ms prior to the presentation of the target word (e.g., warm) and stayed there until the participants responded to the target word. The participants were required to discriminate the target word as rapidly as possible (i.e., warm or cold). We assessed crossmodal priming using a vocal response (cf. [20] for a similar approach). The participants were instructed to speak loudly into a pen microphone attached to their collar. When they spoke, the activation of the voice key terminated the trial and the RT was automatically entered into a data file. The experimenter noted whether or not each participant had responded correctly (e.g., saying “warm” in response to the warm stimulus).
2. *Colour discrimination task.* The colour discrimination task paralleled the thermal word discrimination task except that this time the thermal word was the prime and the colour patch the target.

#### **Experiment 2B: Physical thermal stimuli**

##### **Participants**

Seventeen participants (12 females) took part in this experiment. The participants had a mean age of 36.4 years (*SD* = 12.7 years, range 19–61 years). All of the participants had normal or corrected-to-normal vision and none reported any motor or neurological abnormalities. The experiment was approved by, and carried out in accordance with the regulations of, the Monash University Human Research Ethics Committee. The participants gave their written informed consent before starting the experiment.

##### **Apparatus and stimuli**

This experiment was conducted at Monash University, Australia. We used two thermal temperature stimuli; 34.2°C for the warm stimuli and 17.3°C for the cold stimuli. The thermal stimuli were generated by a *Powertech*™ MP 3081 DC power supply and presented via a solid-state 30×30×4.7 mm CP1.4-71-10L series thermoelectric cooler, commonly known as a Peltier device which, at baseline, has a temperature approximately equal to room temperature (i.e., 22°C). This device functions within the limits of 3.90 Amps, 18.70 Watts and 8.60 Volts. Upon application of a DC current to the Peltier device, heat is transferred across it creating opposing cool and warm sides. Power supply amperage was used to regulate the rise time of the device.

The visual stimuli consisted of 5.1 cm×5.1 cm blue and red squares in RGB colour space (blue squares: hue angle 240°, saturation 85%, and lightness 85%; red squares: hue angle 0°, saturation 85%, and lightness 85%). The squares were presented centrally against a gray background (hue angle 288°, saturation 0%, and lightness 37%) on a 35 cm colour monitor.

##### **Procedure**

Eight experimental trials were created by pairing temperature stimuli (warm and cold), colour stimuli (red and blue) and target modality (temperature and colour). The order in which the trials were presented was randomized for each participant. However, as in Experiment 2A, the response target to be discriminated was tested in a blocked manner to avoid any confusion about which stimulus the participants had to respond to. The order in which the two blocks were presented was counterbalanced across participants. Incorrect responses were disregarded and the pairing in question was presented again at a random point during the remaining trials. A typical session lasted for approximately 15 min, and participants received feedback at the end of the experiment.

1. *Temperature discrimination task.* The participants were seated approximately 45 cm from, and directly in front of, a computer monitor. After a trial was initiated, the participants were given a 2,000 ms on-screen countdown. The participants were then presented with a priming colour patch for a further 2,000 ms prior to placing an index finger on the Peltier device, at which point the target thermal stimulus was presented. The participants were required to discriminate the temperature as rapidly as possible by making a verbal response for temperature (i.e., warm or cold). The participants were instructed to speak loudly into a microphone positioned in front of them. When they spoke, the activation of the voice key terminated the trial and the RT was automatically entered into a data file. The experimenter noted whether or not each participant had responded correctly (e.g., saying “warm” in response to a warm stimulus). The participants were given a randomly-selected practice trial without a coloured square before the experiment proper. The data from the practice trial were not analyzed.
2. *Colour discrimination task.* The colour discrimination task paralleled the temperature discrimination task except for the following. The participants rested an index finger on the Peltier device before the start of each trial; they were allowed to remove their finger from the Peltier device once a response had been made. Each trial was preceded by a four sec on-screen countdown, the purpose of which was to allow the Peltier device to reach a warm (34.2°C) or cool (17.3°C) temperature. A pilot study indicated that it took ∼2,250 ms for participants to notice the ‘warm’ temperature and ∼2,000 ms for them to notice the ‘cold’ temperature. Thus, the 4,000 ms countdown allowed sufficient time for the stimuli to reach a clearly suprathreshold level. After the 4,000 ms countdown, a coloured stimulus (target) appeared on the screen. The participants were required to discriminate the colour as rapidly as possible by making a verbal response identifying the name of the colour (i.e., red or blue). The experimenter noted whether or not each participant had responded correctly (e.g., said “red” in response to a red stimulus). The colour classification block was preceded by a randomly selected practice trial without thermal stimulation. The data from the practice trial were not analyzed.

### Results

#### Experiment 2A: Thermal word stimuli

**Figure 3:**
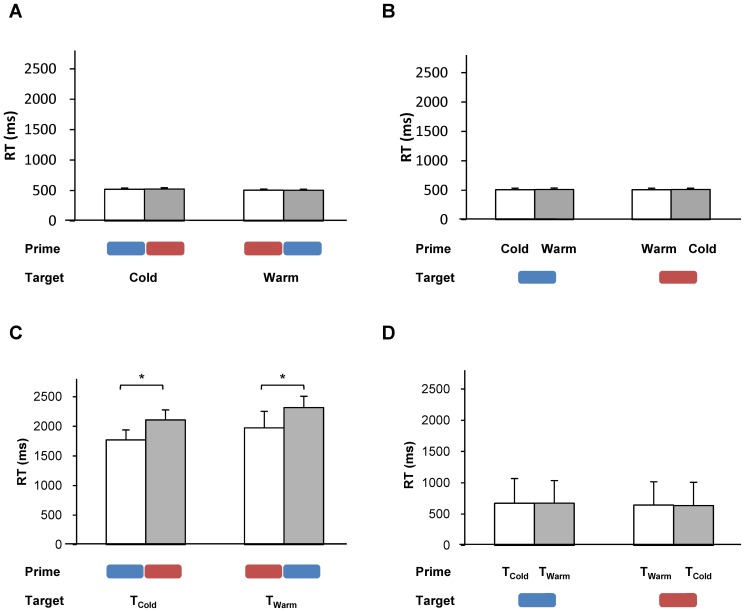
Results of the priming task. RTs for participants discriminating (A) thermal words and (B) colour stimuli in Experiment 2A, and (C) physical temperature and (D) colour stimuli in Experiment 2B. Congruent and incongruent combinations are shown in white and gray, respectively. Error bars denote the standard errors of the means. * indicate statistical significance of *p*<.05 (paired one-tailed *t* test: A one-tailed test is justified in this case given our prediction that the congruent combinations would give rise to shorter RTs than the incongruent combinations).

Group mean RTs when discriminating the thermal word (warm and cold) are shown in Figure 3A. Using RT as a dependent variable, we conducted a repeated-measures ANOVA with the congruency of the prime (congruent/incongruent) and target word (warm/cold) as the within-participants factors. Neither of the main effects were significant [for congruency, *F*(1,13)<1, *n.s.*; for target word, *F*(1,13) = 2.48, *p* = 0.14]. Figure 3B represents group mean RTs when discriminating the colour patch: Red and blue. Using RT as the dependent variable, we conducted the same analysis with the congruency of the prime and the colour of the target as the within-participants factors. The main effects of prime congruency and type of colour were not significant [for congruency, *F*(1,13) = 1.64, *p* = 0.22; for colour type, *F*(1,13)<1, *n.s.*]. These results indicate that the crossmodal correspondences between colour and temperature did not give rise to a significant influence on RTs when discriminating the thermal words or colour patches in a simple priming task.

#### Experiment 2B: Physical temperature stimuli

Group mean RTs for judging the type of thermal stimulations (warm and cold) are presented in Figure 3C. Using RTs as a dependent variable, a repeated-measures ANOVA was conducted with the congruency of the prime (congruent/incongruent), the type of target (red/blue or warm/cool), and target modality (colour/temperature) as within-participants factors. The main effect of target modality was significant [*F*(1,16) = 80.06, *p*<.001]; the RT to discriminate the stimulus pair was shorter when identifying the colour than when identifying the temperature. The main effect of prime congruency was significant [*F*(1,16) = 9.06, *p = *.008]; the RT to discriminate the temperature of the target was shorter with congruent combinations of prime and target (red-warm, blue-cold) than with incongruent combinations (red-cool, blue-warm). The main effect of target type was not significant [*F*(1,16)<1, *n.s.*]. The interaction between target modality and congruency was significant [*F*(1,16) = 12.37, *p = *.003]. Post-hoc comparisons established that the RT to discriminate the temperature of the target was shorter with congruent combinations of prime and target (red-warm, blue-cold) than with incongruent combinations (red-cool, blue-warm), but congruency had no effect on participants’ RTs when they were required to discriminate the colour of the target. Figure 3D represents the group mean RTs when discriminating the colour of the patches.

Taken together, the results of Experiment 2 indicate that the crossmodal correspondence between colour and temperature modulates RTs when participants have to discriminate physical thermal stimulation (Figure 3C), but has no effect when people are asked to discriminate thermal words (Figure 3A) or colour stimuli (Figures 3B and 3D).

## Discussion

Experiment 1 was designed to assess the strength of the association between colour and temperature using a simplified version of the IAT. The results demonstrated that the congruent assignment of the stimuli to the response keys (red-warm, blue-cold) gave rise to shorter RTs than the incongruent key assignments (red-cold, blue-warm), regardless of whether the response target was a thermal word or a colour patch. These results therefore indicate that the red-warm and blue-cold congruency effect holds at the level of response selection, indicating that the speed of response choice to a colour or thermal stimulus is influenced by the colour-temperature correspondences. To the best of our knowledge, this is the first study to have established the crossmodal correspondence between colour (hue) and temperature using an objective performance measure. In previous studies, colour-temperature correspondences were demonstrated using a variety of subjective measures [4]–[6], [11]–[13], [21]. Our findings, along with the results from those studies, confirm the existence of the effects of colour-temperature correspondences.

The results of Experiment 2 demonstrate that this colour-temperature correspondence has no effect on the RTs to discriminate the colour of a stimulus, but can affect the RTs required to discriminate a thermal stimulus. This asymmetrical effect suggests that the colour-temperature correspondence might be unidirectional, which is not unexpected given that colours are often used to indicate temperature, whereas temperature is seldom (if ever) used to indicate colour. A similar unidirectional effect is observed in synaesthetes who often perceive colours that have been induced by other kinds of sensory stimuli (e.g., by sounds). Although it would be unwise to assume a direct relationship between synaesthetes and crossmodal correspondences in non-synaesthetes [22], there may be some common underlying neural mechanisms [23]. Moreover, a recent study reported that the strength of correspondence in one direction is not necessarily the same as the strength of the association when probed in the opposite direction [24]. Specifically, these authors found that participants were more likely to agree that there was an association between colour and another sensory dimension (i.e., pitch) when they were asked about the plausibility of the association in the direction of sound pitch→colour, rather than when asked about colour→sound pitch.

That said, it should also be noted that the effect of colour-temperature correspondences also depends on the type of thermal stimulus under consideration. When the target was a thermal word, there was no effect. However, when the target was a physical thermal stimulus, RTs were significantly reduced. The difference between the results for a thermal word target and a physical thermal target might therefore illustrate some kind of modality dominance. Specifically, visual information (i.e., a colour patch) might dominate over tactile information regarding temperature that may take longer to process, but has no effect on the semantic information that has a comparable processing time. It is, however, more likely that the results reflect differences in the strength of the link between the various target stimuli and responses. When a thermal stimulus was the target, the participants’ task was to classify the thermal word or the physical thermal stimulus as belonging to a warm or cold category. The thermal words presumably have strong linguistic links to the response category. Consequently, the RT is short (500–600 ms) and can hardly be affected by the colour prime, regardless of its congruency relative to the target. On the other hand, when the target consisted of a physical thermal stimulus, it might have been harder for our participants to establish the categorical and/or linguistic links between the physical temperature and the concepts of warm or cold, as reflected by the long RTs (∼2,000 ms). In this case, according to the Semantic Coding Hypothesis, dissimilar types of signals in different sensory modalities (e.g., colour and physical temperature) can interact with each other at a post-perceptual level of information processing (e.g., [25]). The colour-temperature combination that is congruent with the post-perceptual association (i.e., the red-warm and blue-cold congruencies as confirmed by the results of the IAT) might, then, facilitate a participant’s classifying/labeling a relatively low temperature as cold and a relatively high temperature as warm. As a result, the RT to the physical thermal stimulus can be reduced when the colour of the prime is deemed congruent. Taken together, the results of the present study suggest that the effect of colour-temperature correspondences on human information processing are more effective when the stimuli have a weak connection to the response category, and/or take a relatively long time to be processed.

In Experiment 2, the stimulus onset asynchrony (SOA) between a colour and a thermal word was 2,000 ms and we found no priming effect. This SOA is relatively longer than those typically used in the semantic priming experiments (∼200 ms), and was used because we wanted the interval to be consistent with the one used with the physical thermal stimulus in order to allow for direct comparison between these two types of thermal stimulus. It has been suggested that short SOAs (∼200 ms) typically involve automatic, unconscious processing, while the long SOAs (>1,000 ms) primarily involve conscious processing, such as expectancy and matching [26]. Although the underlying processing may be different for the two, short and long SOAs typically give rise to similar priming effects [27]. Our present results suggest that there is no priming effect for thermal words under conscious processing. However, whether the priming effect exists for unconscious processing needs further investigation with short SOAs.

The correspondences between colour and temperature and the processing preference for the congruent combination of stimuli revealed by the results of the IAT are presumably based on the internalization of the correlations between stimuli/dimensions that are present in the environment, and which are likely learnt through experience [16], [28]–[30]. In the case of natural correlations, for example, fire and the sun are both warm; hence all the colours of fire and the sun might be associated with warmth. Water and forests are cool; hence blue and green may be associated with coolness. The association might also be linked to the fact that our skin gets redder when we are warm and blue when we are very cold [31]. That said, it has been argued that many other crossmodal correspondences are based on the internalization of the natural correlations present in the environment including pitch–elevation, pitch–size, and loudness–size correspondences (see [16] for a review).

In those cases where the crossmodal association is learnt through experience, different cultures and environments could possibly influence the colour-temperature association. Notably, however, in the present study we obtained consistent results for the colour-temperature association despite the fact that our experiments were conducted in two different cultural environments (Experiment 1 & 2A in Japan, and 2B in Australia). It is presumably because the colour-temperature association cues in Japan are the same as those in Australia that such a similar pattern of results were obtained. Bathroom/kitchen taps in Japan are labeled with red and blue, which symbolize hot and cold, respectively. Thus, as Westerners, Japanese people are also exposed to multiple stimuli on a daily basis that reinforce the colour-temperature association. As an example, it has been reported that in some African countries, the association is reversed, with blue signifying hot and red cold [7]. In this case, an IAT might reveal a different pattern of association and processing preference to those documented here. As such, it is possible, as Morgan *et al*. [10] argued, that these results reflect the idea that conventional colour/temperature correspondences are based on “loosely held cultural norms” (p. 125). This view is consistent with that of Ernst [33] who showed that participants could be trained to integrate unrelated stimuli, and thus the integration of sensory signals is certainly not hardwired. Nevertheless, the correspondences between colour and temperature, and between other stimulus features in different sensory modalities, can be regarded as an important means used by which our brains determine which sensory signals from different sensory modalities to integrate. Further, these correspondences hint at how humans efficiently process the information that arrives from multiple senses at the same time [16], [25], [34].

## Conclusions

In conclusion, the present study was designed to determine whether the impact of colour-temperature correspondences would be different at two distinct levels of information processing. The results of the IAT confirmed that the association exists at the level of response selection, thus indicating that a participant’s responses to colour or thermal stimuli are influenced by the crossmodal correspondence between colour and temperature. The priming experiment measures one’s ability to selectively attend to one source of sensory information while ignoring the influence of the other stimulus presented somewhat earlier in time. The results of the priming experiment revealed that priming a colour stimulus affected thermal discrimination RTs, but thermal cues did not influence colour discrimination responses. These findings are consistent with those from studies that have used subjective measures and confirm the existence of the effects of colour-temperature correspondences.
